# 

**Published:** 1858

**Authors:** 


### CONTENTS, No. 3, Vol. IV.

ORIGINAL COMMUNICATIONS. Page.

Inexactness of Medical Writers, A. S. Henderson, M. D. 162
Air Tubercles from the Living Subject, " " 163
Extra Fingers and Toes, Wm Gutch, M. D. - - - - 165
Surgical Cases, W. R. Marsh, M. D. - - - - - 166
Cases of Practice, D. L. McGugin, M. D. - - - - - 171

DEPARTMENT OF SELECTIONS.

Loss of nearly half the bones of skull, with exposure and sloughing of portion of the Brain, - - - - 182
Pathology and Treatment of Aroemia - - - - 186
Cases of Sciatica cured with Strychnine, - - - - 193
Lecture on frequent Micturation, - - - - - 195
The Solvent process of Glycerin, - - - - - - 208
Effect of Cold Water on the Circulation, - - - 209
Ætiology of Diabetes, - - - - - - - 212
Hydranlic Improvements, - - - - - - 216

EDITORIAL DEPARTMENT.

Professional Secresy, - - - - - - - 217
Philadelphia Hospital, - - - - - - - 222
Bust of Prof. Mussy, - - - - - - - 224
Effects of Intermarriage, - - - - - - - 226
Medical Works, - - - - - - - - 227
Excito-Secretory System, - - - - - - - 228
Books Received, - - - - - - - - 229
Iowa Medical Journal, - - - - - - - 231
College Matters, Telescope, N. W. Review, Hard Times, - 232
Eighth Annual Announcement of Medical Department Iowa University, - . - - - - - - - 233

